# The Application of Soft Robotic Gloves in Stroke Patients: A Systematic Review and Meta-Analysis of Randomized Controlled Trials

**DOI:** 10.3390/brainsci13060900

**Published:** 2023-06-02

**Authors:** Ming-Jian Ko, Ya-Chi Chuang, Liang-Jun Ou-Yang, Yuan-Yang Cheng, Yu-Lin Tsai, Yu-Chun Lee

**Affiliations:** 1 Department of Education, Taichung Veterans General Hospital, Taichung 407219, Taiwan; albert870205@gmail.com; 2 Department of Physical Medicine and Rehabilitation, Taichung Veterans General Hospital, Taichung 407219, Taiwan; y065e225@vghtc.gov.tw (Y.-C.C.); s851075@ym.edu.tw (Y.-Y.C.); 3 Department of Physical Medicine and Rehabilitation, Chang Gung Memorial Hospital, Linkou, Taoyuan 333423, Taiwan; ohyoung18287@gmail.com; 4 Department of Post-Baccalaureate Medicine, College of Medicine, National Chung Hsing University, Taichung 402202, Taiwan; 5 Department of Exercise Health Science, National Taiwan University of Sport, Taichung 404401, Taiwan; 6 Department of Industrial Engineering and Enterprise Information, Tunghai University, Taichung 407224, Taiwan

**Keywords:** soft robotic glove, stroke, rehabilitation, hemiparesis, meta-analysis

## Abstract

Wearable robotic devices have been strongly put into use in both the clinical and research fields of stroke rehabilitation over the past decades. This study aimed to explore the effectiveness of soft robotic gloves (SRGs) towards improving the motor recovery and functional abilities in patients with post-stroke hemiparesis. Five major bibliographic databases, PubMed, Embase, Cochrane Library, Web of Science, and the Physiotherapy Evidence Database, were all reviewed for enrollment regarding comparative trials prior to 7 March 2023. We included adults with stroke and compared their rehabilitation using SRGs to conventional rehabilitation (CR) on hand function in terms of the Fugl-Meyer Upper Extremity Motor Assessment (FMA-UE), Fugl-Meyer Distal Upper Extremity Motor Assessment (FMA-distal UE), box and blocks test score, grip strength test, and the Jebsen–Taylor hand function test (JTT). A total of 8 studies, comprising 309 participants, were included in the analysis. Compared to CR, rehabilitation involving SRGs achieved better FMA-UE (MD 6.52, 95% CI: 3.65~9.39), FMA-distal UE (MD 3.27, 95% CI: 1.50~5.04), and JJT (MD 13.34, CI: 5.16~21.53) results. Subgroup analysis showed that stroke latency of more than 6 months and training for more than 30 min offered a better effect as well. In conclusion, for patients with stroke, rehabilitation using SRGs is recommended to promote the functional abilities of the upper extremities.

## 1. Introduction

According to the World Stroke Organization, stroke remains the second-leading cause of death and the third-leading cause of death and disability combined in the world [1]. Chronic dysfunction affects 60% of the affected individuals, and of those, 60–80% experience functional dyskinesia in their upper extremities [2]. 

Developments in the use of robotic devices have shown promise in aiding hand functional recovery [3]. However, previous exoskeleton devices have always presented significant drawbacks due to their heavy and bulky structures, limited range of motion in human joints, and their unaesthetic appearance. Robotic gloves have since emerged as a more compact and intuitive alternative to exoskeletons. These glove-like devices envelop the paretic hand, providing a more comfortable and convenient solution [4] for overcoming a patient’s condition. Other advantages of soft robotic wearable devices as compared to exoskeleton devices include maintaining the wearer’s mobility and flexibility without over-constraining the joints, less time wearing the device due to there being no need for precise joint alignment, being more comfortable to don and doff (meaning easier to put on and remove) and improving portability due to their reduced overall weight [5]. 

There are several methods regarding clinical evaluation for those experiencing post-stroke motor function disability. The Fugl-Meyer Assessment (FMA) is a well-designed, feasible, and efficient clinical examination method that has been tested widely in the stroke population. Its primary value is the 100-point motor domain, which has received the most extensive evaluation. Additionally, the method is also responsive to changes in motor impairment following stroke [6]. Another tool that has been widely used in clinical and research settings is the Jebsen-Taylor hand function test (JTT). This test involves seven subsets within the test whom represent a spectrum of hand function, with the patient’s performance in each subset timed and compared with the established norms [7]. The box and block test (BBT) is also reliable and valid for patients with stroke as it is used to measure gross manual dexterity. This test measures the number of 1-inch blocks a patient can transport from one box to its adjacent box within 60 s. The greater the number of blocks per minute, the better the performance of gross manual dexterity [8]. Additionally, maximal grip strength measurement is also a great tool which can easily quantify one’s weaknesses and recovery following a stroke, and has proven to be reliable in both asymptomatic and symptomatic subjects [9]. All of these measurement tools are capable of providing objective methods to help assess patients and improve their clinical outcomes when diagnosed with hemiparesis.

Over the years, several studies have evaluated the effectiveness of robotic devices on stroke patients, but few of them have confined themselves to only the use of the soft robotic glove (SRG). In recent years, Fardipour et al. and Hernández Echarren et al. have each published systematic reviews regarding the therapeutic effects of wearable robotic gloves on hand function in stroke patients [4,10]. Nevertheless, neither of them involved trials that were all randomized controlled trials and completely focused on SRGs. Therefore, the purpose of this study was to conduct a comprehensive meta-analysis in order to obtain objective outcomes, as well as thoroughly discuss the clinical application of SRGs in stroke patients.

## 2. Materials and Methods

### 2.1. Search Strategy and Selection Criteria

The protocol for this review was registered in the International Prospective Register of Systematic Reviews (PROSPERO CRD42023387935). This study was performed in accordance with the Preferred Reporting Items for Systematic Reviews and Meta-analyses (PRISMA) 2020 statement [11] shown in Table S1. Two investigators (K-MJ and T-YL) performed the initial literature screening by reviewing titles and abstracts in five electronic databases (PubMed, Embase, Cochrane Library, Web of Science, and the Physiotherapy Evidence Database (PEDro)) prior to March 7, 2023, without applying any filters. A manual literature search of bibliographies from the retrieved articles and published reviews for eligible publications was also performed. The following keywords and their synonyms were applied to identify relevant publications: “soft robotic glove”, ‘‘soft wearable robot”, and ‘‘stroke’’. A detailed description of the search strategy is provided in Table S2.

We included randomized control trials (RCTs) if they met the following criteria: (1) Population: patients with post-stroke hemiparesis (PSH) who had received or were scheduled to receive rehabilitation; (2) Intervention: rehabilitation programs involving SRGs or other similar devices; (3) Control: conventional rehabilitation (CR) programs, such as physical therapy and occupational therapy; (4) Outcomes: including Fugl-Meyer Upper Extremity Motor Assessment (FMA-UE), Fugl-Meyer Distal Upper Extremity Motor Assessment (FMA-distal UE), grip strength, BBT, and JTT score. Studies were excluded if their data were inaccessible. SRGs were defined as compact and wearable devices but not rigid exoskeleton devices. Participants in the control group received rehabilitation without the use of SRGs or any similar device. Any discrepancies were discussed with a third investigator (C-YC) in order to reach a consensus.

### 2.2. Outcome Measures

Primary outcomes were determined by FMA-UE and FMA-distal UE scores, while secondary outcomes were based on grip strength, BBT, and JTT scores. The patient’s grip strength was recorded in pounds (lbs).

### 2.3. Data Extraction and Quality Assessment

Two investigators (K-MJ and T-YL) independently screened potential titles and abstracts for eligibility. Subsequently, the full text of each potentially eligible article was assessed. All discrepancies were discussed and resolved in consultation with a third investigator (C-YC). The following variables were extracted: participant characteristics, outcome measurements, follow-up period, and intervention protocol (type of device, training content, frequency, training length, and total training duration). We also contacted the authors for details when data were missing and excluded studies from data analysis when their data were inaccessible or the authors did not respond.

Two investigators (C-YC and K-MJ) independently evaluated the risk of bias for all studies and assessed the quality of the articles included in the analysis using Version 2 of the Cochrane tool to assess the risk of bias in randomized trials (RoB 2.0 tool) [12]. Conflicting opinions were discussed until a consensus was reached, with a third investigator (T-YL) being consulted when necessary.

### 2.4. Data Synthesis and Statistical Analysis

The results were analyzed using Review Manager V.5.4 software (Cochrane Collaboration, London, UK). Continuous data were expressed as mean ± standard deviation (SD) and summarized as a standardized mean difference (MD) with 95% confidence intervals (CIs). A random effects model was used to assess the pooled estimated effect of the intervention. Subgroup analyses were conducted based on stroke latency, type of device, training length, and total training duration in order to explore the immediate therapeutic effects of SRGs. The heterogeneity of the outcome measures was examined using the Cochrane *I*^2^ statistic and Cochran’s Q test. In cases of statistically significant heterogeneity—defined as *I*^2^ > 75% and Cochran’s Q test *p* < 0.05—a sensitivity analysis was performed to explore the possible cause of the heterogeneity. A funnel plot and the Egger regression test were conducted to assess publications bias, and a two-tailed *p*-value lower than 0.1 was regarded as statistically significant. Egger regression test results were analyzed using comprehensive meta-analysis (CMA 3.0). The Grading of Recommendations Assessment, Development and Evaluation (GRADE) framework was adopted in order to evaluate the certainty of evidence from the included trials [13].

## 3. Results

### 3.1. Study Selection and Characteristics

**Figure 1:**
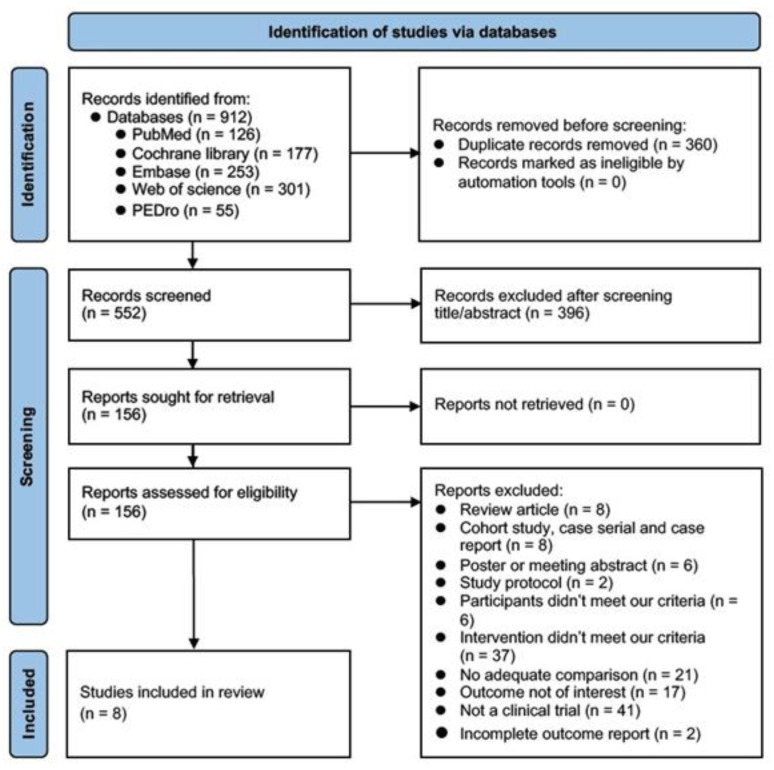
PRISMA2020 flow chart showing the literature search and selection process.

Our electronic search initially yielded a total of 912 studies. After primary screening we identified 156 articles for use in our full-text assessment. Ultimately, eight studies were incorporated into our analysis involving a total of 309 participants after assessment for eligibility [14,15,16,17,18,19,20,21]. The flowchart of the selection procedure is shown in Figure 1. The reasons for exclusion are shown in Table S3.

A total of eight randomized controlled studies were included in this meta-analysis. Three studies introduced a rehabilitation program consisting of wearing the RAPAEL Smart Glove [15,17,19]. There were a total of 142 patients who received therapy with SRGs and 134 patients who received CR. The characteristics of these studies are summarized in Table 1, with each study’s SRG protocols summarized in Table 2. Six trials measured FMA-UE [14,15,17,19,20,21], three calculated FMA-distal UE [17,19,20], four recorded JTT scores [15,17,18,19], three examined grip strength [16,18,19], while two studies examined BBT scores [19,21]. The total training duration ranged from two to four weeks.

### 3.2. Methodological Quality of Included Trials

**Figure 2:**
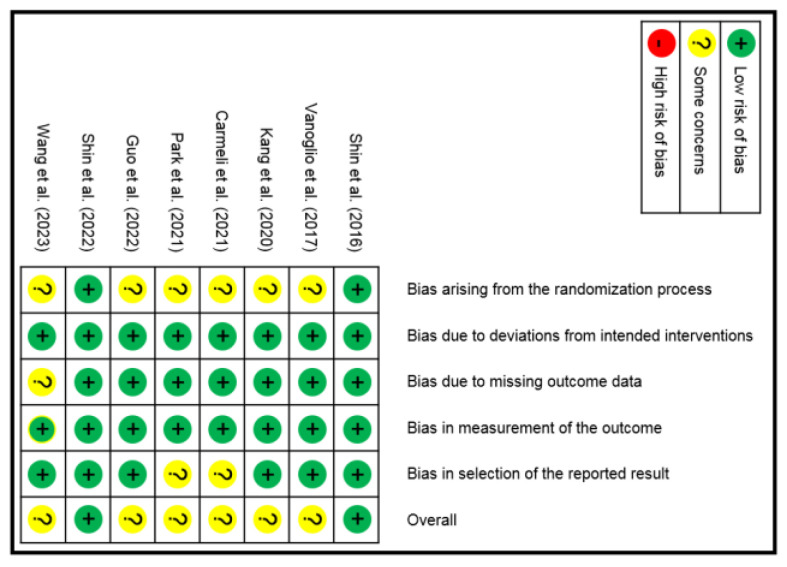
Risk of bias summary for the included trials based on RoB 2.0. Shin et al., 2016 [17]; Vangolio et al., 2017 [16]; Kang et al., 2020 [19]; Carmeli et al., 2021 [21]; Park et al., 2021 [18]; Guo et al., 2022 [20]; Shin et al., 2022 [15]; and Wang et al., 2023 [14].

According to the RoB 2.0, two RCTs [15,17] were considered to have a low risk of bias, while the other six [14,16,18,19,20,21] were rated as having some concerns. A summary of the risk of bias is shown in Figure 2. The GRADE framework was introduced for intergroup outcome measure comparison and is presented in Table S4.

### 3.3. Effects of Intervention

#### 3.3.1. Primary Outcome: FMA-UE Scores

**Figure 3:**
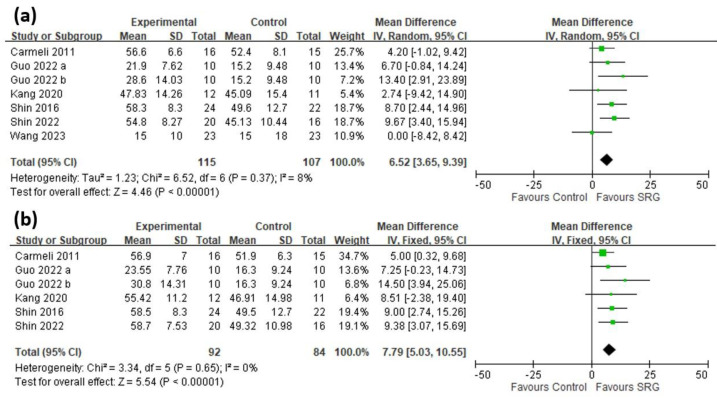
Mean difference (95% CI) of the immediate (**a**) and long-term (**b**) effect of SRGs on FMA-UE compared with CR [14,15,17,19,20,21]. Guo 2022 a used a steady-state visually evoked potentials-based brain computer interface soft robotic glove [20]. Guo 2022 b used a computer-controlled soft robotic glove [20]. (SRG: soft robotic glove, FMA: Fugl-Meyer Assessment scores, UE: upper extremity, CR: conventional rehabilitation.)

A total of 7 trials [14,15,17,19,20,21] involving 222 patients were included in the quantitative analysis (Figure 3). A significant improvement in FMA-UE scores was demonstrated in those patients receiving therapy with SRGs through the assessment which was made immediately after the intervention (MD 6.52, 95% CI: 3.65~9.39, *I*^2^ = 8%). A total of 6 trials [15,17,19,20,21] involving 176 patients demonstrated the follow-up assessment, which also revealed a significant improvement in FMA-UE scores (MD 7.79, 95% CI: 5.03~10.55, *I*^2^ = 0%) (Figure 3), with the funnel plot shown in Figure S1. In subgroup analyses, patients who had reached chronic stroke status (latency >6 months) showed significant improvement in their FMA-UE score (MD 4.93, 95% CI: 0.93~8.93, *I*^2^ = 19%), with those whose stroke latency was less than six months also showing significant improvement (MD 8.84, 95% CI: 4.47~13.22, *I*^2^ = 0%). The three trials [15,17,19] which used the RAPAEL Smart Glove revealed significant improvement in FMA-UE scores (MD 8.43, 95% CI: 4.27~12.59, *I*^2^ = 0%), as did the other four studies which involved other devices (MD 5.29, 95% CI: 0.90~9.67, *I*^2^ = 27%). In the subgroup involving a training length of less than 30 min, significant improvement in FMA-UE scores was found (MD 5.85, 95% CI: 2.49~9.21, *I*^2^ = 15%), with those trials whose training length was more than 30 min also showing significant improvement (MD 9.01, 95% CI: 2.77~15.26, *I*^2^ = 3%). Additionally, whether those trials received a total training duration of more than two weeks (MD 7.12, 95% CI: 3.67~10.57, *I*^2^ = 4%) or not (MD 5.51, 95% CI: 0.04~10.98, *I*^2^ = 27%), significant improvements were achieved in both. Further sensitivity analysis was not needed due to a low heterogeneity (*I*^2^ < 50%) being found in all subgroups. The detailed subgroup analysis results are presented in Table 3. The certainty of the evidence ranged from low to moderate according to the GRADE appraisal.

#### 3.3.2. Primary Outcome: FMA-Distal UE Score

**Figure 4:**
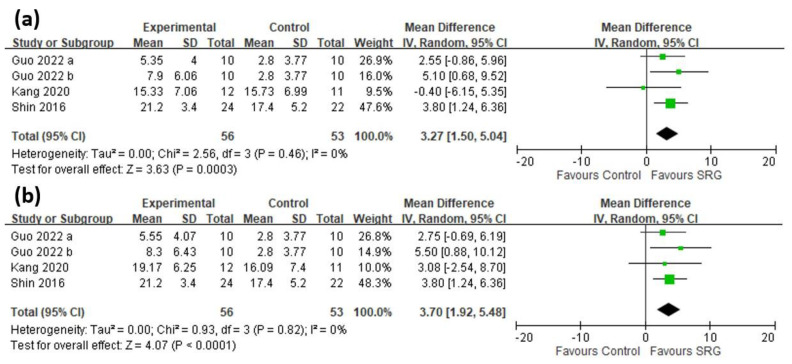
Mean difference (95% CI) of the immediate (**a**) and long-term (**b**) effect of SRGs on FMA-distal UE, compared with CR [17,19,20]. Guo 2022 a used a steady-state visually evoked potentials-based brain computer interface SRG [20]. Guo 2022 b used a computer-controlled soft robotic glove [20]. (SRG: soft robotic glove, FMA: Fugl-Meyer Assessment scores, UE: upper extremity, CR: conventional rehabilitation).

A total of 4 trials [17,19,20] involving 109 patients were included in the quantitative analysis (Figure 4). A significant improvement in FMA-distal UE scores was demonstrated in patients receiving therapy with SRGs no matter whether the assessment was performed immediately after the intervention (MD 3.27, 95% CI: 1.50~5.04, *I*^2^ = 0%) or during the follow-up assessment (MD 3.70, 95% CI: 1.92~5.48, *I*^2^ = 0%) (Figure 4), with the funnel plot shown in Figure S2. In subgroup analyses, the three trials whose patients were designated as chronic stroke status (latency >6 months) showed significant improvement in FMA-distal UE scores (MD 3.66, 95% CI: 1.80~5.52, *I*^2^ = 0%). Two trials [17,19] involving the RAPAEL Smart Glove revealed no significant improvement in FMA-distal UE scores (MD 2.52, 95% CI: −1.27~6.31, *I*^2^ = 42%), while the remaining two trials using other devices did see improvement (MD 3.50, 95% CI: 0.80~6.20, *I*^2^ = 0%). In the subgroup involving those undergoing a training length of more than 30 min, significant improvement in FMA-distal UE scores was found (MD 3.50, 95% CI: 0.80~6.20, *I*^2^ = 0%), while those trials where the training length was less than 30 min showed no significant improvement (MD 2.52, 95% CI: −1.27~6.31, *I*^2^ = 42%). As for subgroup analysis regarding total training duration, significant improvement was found no matter whether the duration was for either more or less than two weeks. Further sensitivity analysis was not required due to low heterogeneity (*I*^2^ < 50%) being found in all subgroups. The detailed subgroup analysis results are presented in Table 3. The certainty of the evidence ranged from low to moderate according to the GRADE appraisal.

#### 3.3.3. Secondary Outcome: JTT Scores, Grip Strength, and BBT Scores

Four studies [15,17,18,19] reported on the effect of therapy involving SRGs when compared to CR on JTT scores, with the results demonstrating both immediate and long-term improvement in a significant manner: (MD 13.34, 95% CI: 5.16–21.53, *I*^2^ = 8%) and (MD 19.38, 95% CI: 9.94–28.82, *I*^2^ = 0%), respectively. The certainty of the evidence was moderate, according to the GRADE appraisal. However, no significant improvement was revealed upon analysis of grip strength (MD 3.11, 95% CI: −6.25~12.47, *I*^2^ = 0%) or BBT scores (MD −0.75, 95% CI: −9.03~7.54, *I*^2^ = 0%) (Figures S3–S5). The certainty of the evidence in each was low, according to the GRADE appraisal.

## 4. Discussion

### 4.1. Summary and Contributions

In this study, we conducted a meta-analysis to investigate the effectiveness of rehabilitation involving SRGs on hand function in stroke patients. Our results show that rehabilitation with SRGs significantly improved FMA-UE scores, FMA-distal UE scores, and JJT scores when compared to only CR, with these improvements being observed not only immediately after the intervention but also in subsequent follow-up assessments. Regarding distal hand function, our findings suggest that chronic stroke patients who received rehabilitation combined with the use of SRGs may experience a better immediate effect, particularly during training sessions lasting more than 30 min. To the best of our knowledge, this study is the first meta-analysis focusing solely on the effect that SRGs have on hand function in stroke patients.

### 4.2. Comparison with Previous Studies

Up until now, few systematic reviews or meta-analyses of randomized controlled trials have discussed the effect that SRGs have on hand function in stroke patients. Fardipour et al. published a systematic review in 2022 investigating the therapeutic effects of wearable robotic gloves on improving hand function in stroke patients. However, use of the device was not confined to SRGs, and the included trials were not all randomized controlled trials. Additionally, no meta-analysis was performed. In another study, Luo et al. published a systemic review and meta-analysis evaluating the synergistic effect of combined mirror therapy on the upper extremities in patients with stroke, with one of the experimental groups going through intervention involving mirror therapy with a mesh glove [22]. Fernández-Vázquez et al. published a systematic review and meta-analysis in 2022, but the study focused on intervention involving Haptic Glove Systems in combination with semi-immersive virtual reality (SVR) for use in upper extremity motor rehabilitation after stroke [23]. The study we have performed was the first systematic review and meta-analysis which has purely discussed the effect of SRGs on stroke patients, with the included studies all being randomized controlled trials.

According to previous meta-analysis, we have found similarities in several outcomes, but there were also differences which remained in some of the results. The meta-analysis published by Fernández-Vázquez et al. evaluates the random effect that gloves and SVR have on FMA, JTT, and BBT scores, revealing that the combined use of rehabilitation gloves with SVR produces significant improvements over the use of only CR treatment in the upper extremity functions of stroke patients in both the short and long term, regardless of whether or not associated CR is also performed. As for our study, we precisely analyzed FMA, JTT, and BBT scores, respectively, and found significant improvements in FMA -UE scores, FMA-distal UE scores, and JJT scores. However, no significant improvement in BBT scores was seen in our study. With regards to grip strength, both Fernández-Vázquez et al. and our study revealed no significant improvement over simply using CR.

### 4.3. Clinical Effect

Concerning the minimal clinically important difference (MCID), this often varies across patient populations and post-onset periods. Thus, it is necessary to have evidence of MCID at each post-onset period and each level of paresis [24]. The recovery time after a stroke is often divided into phases. The Stroke Roundtable Consortium has proposed designating the first 7 days as the acute phase, the first 6 months as the subacute phase, and from 6 months onwards as the chronic phase [25]. The estimated MCID score for upper extremity motor recovery among patients with subacute stroke is 9 to 10 for FMA-UE scores [26]. Therefore, according to our analysis, four studies included patients in the subacute stroke phase. The mean of the increased amount in FMA-UE scores was 10.321 in the soft robotic group but only 4.653 in the control group, which demonstrates that a rehabilitation program involving intervention with SRGs can achieve meaningful clinical improvements in upper extremity motor recovery among subacute stroke patients (Figures S6–S8). Alternatively, the estimated MCID score for upper extremity motor recovery among patients with chronic stroke is 4.25 to 7.25 for FMA-UE scores [24]. Regarding our analysis, three trials included patients in the chronic stroke phase. The mean of the increased amount in FMA-UE scores was 7.377 in the soft robotic glove group but only 1.114 in the control group, which reveals that a rehabilitation program involving intervention with SRGs can also achieve meaningful clinical improvement in upper extremity motor recovery among chronic stroke patients (Figures S6–S8). Conclusively, when compared with the control group, intervention using SRGs can achieve MCID in both subacute and chronic stroke patients.

Regarding proprioception for the orchestration of muscles to better perform targeted motions, biofeedback plays a critical role. Biofeedback can provide the patient with immediate and accurate feedback on messages regarding one’s body function by taking intrinsic physiological signals and making them extrinsic. During biofeedback, patients would be connected to electrical sensors which allow medical personnel to help receive information about a patient’s body. This technique gains even further significance for its use in the rehabilitation of neurological disorders such as stroke, requiring compensation of motor and sensory functions which may be augmented by biofeedback devices [27]. Most of the soft robotic devices adopted in the trials that we have included here did contain a biofeedback system which could be used in the form of either electromyography which measures muscle tension or electroencephalography which measures brain wave activity. The influences of biofeedback content on robotic post-stroke gait rehabilitation have been studied extensively. A systematic review published by Stanton et al. in 2017 reveals that biofeedback improves performance in lower limb activities more than simply the use of typical therapy in people following stroke [28]. We believe that the improvements SRGs make on hand function are also strongly associated with a biofeedback system.

### 4.4. Subgroup Analysis

With regards to subgroup analysis, we were impressed by the more significant improvements made in the distal extremities by patients in the chronic stroke phase than those made in the subacute phase. It has become well known that neuroplasticity plays an important role towards improving one’s condition after people experience injuries such as stroke or traumatic brain injury. Neuroplasticity is defined as the ability of the nervous system to change its activity in response to intrinsic or extrinsic stimuli by reorganizing its structure, functions, and connections [29]. A previous study has shown that neuroplasticity was most prominent shortly after stroke, particularly during the first thirty days of the post-stroke period, before diminishing over subsequent sessions [30]. We believe that even CR without the use of SRGs could help achieve improvement to some extent for stoke patients in the subacute phase due to neuroplasticity remaining strong. However, since neuroplasticity diminishes gradually over time after stroke, the superiority of the soft robotic glove group over the control group was more obviously seen among stroke patients in the chronic phase. Furthermore, we found that when compared with total training duration, training length had a more positive influence on hand function. The subgroup analysis of FMA-distal UE scores revealed that significant improvement was made only when the training length lasted for more than 30 min per session. This result is reasonable considering that a longer training length would likely have a greater effect on any improvement regarding fine tuning the motor skills of the distal extremities. However, the most suitable training duration involving SRGs for stroke patients remains uncertain, and thus, further research for evaluation of this variable remains necessary.

### 4.5. Limitations

However, several limitations still exist. Firstly, only a few related trials which completely fulfill our inclusion criteria currently exist, and most of them have been published in an Asian country. Furthermore, it was not only the design of the rehabilitation program and the soft robotic device which were both adopted by each trial that were different, but it was also the long-term follow-up period which was diverse among the different studies that we had included. Additionally, the training period in the control group was prolonged in order to fill the time taken by the SRG training sessions used in several studies [14,15,16,17,19,20,21], which may have caused heterogeneity between the different studies.

### 4.6. Future Work

To better demonstrate a more comprehensive result, further studies are required in the future in order to maintain consistency in the design of SRGs, the period of each training session, the total training duration, and the follow-up period. These studies should be performed in order to better help achieve a more complete analysis.

## 5. Conclusions

Our results support the immediate and long-term effectiveness of conventional rehabilitation combined with SRGs in promoting the functions of extremities in patients with PSH, based on improvements seen in FMA-UE, FMA-distal UE, and JTT scores. The effect on distal hand function was most significant when rehabilitation occurred which consisted of SRG use exceeding 30 min per session and when the latency of the stroke was more than six months. These findings offer a perspective on refined SRG prescriptions for patients experiencing PSH. Future randomized controlled trials involving more varied stroke patients and a uniform prescription are still needed in order to better explore the effects of SRGs.

## Figures and Tables

**Table 1:** Characteristics of the included studies.

Study	Design	Location	Participants		Intervention	Outcome Measures	Follow-Up Period
Carmeli et al. (2011) [21]	RCT	Israel	Mean age = 60 years Mean stroke latency = 10 days Stroke type: IS (87%), HS Affected arm, right: 52%	N = 31	Exp = SRG + PT + OT Con = PT + OT	FMA-UE, BBT	1, 3, 4 weeks
Shin et al. (2016) [17]	RCT	Korea	Mean age = 58 years Mean stroke latency = 14 months Type of stroke: IS (63%), HS Affected arm, right: 44%	N = 46 ^a^	Exp = SRG + OT Con = OT	FMA-UE, FMA-distal UE, JTT	2, 4, 8 weeks
Vanoglio et al. (2016) [16]	RCT	Italy	Mean age = 73 years Mean stroke latency = 17 days Stroke type: IS(63%), HS Affected arm, right: 30%	N = 30	Exp = SRG Con = PT	Grip strength	6 weeks
Kang el al. (2020) [19]	RCT	Korea	Mean age = 57 years Mean stroke latency = 25 days Stroke type: IS (35%), HS Affected arm, right: 52%	N = 23 ^b^	Exp = SRG + OT Con = OT + Self-training	FMA-UE, FMA-distal UE, BBT, JTT, Grip strength	2, 6 weeks
Park et al. (2021) [18]	RCT	Korea	Mean age = 61 years Mean stroke latency = ≤1 month Stroke type: N/S Affected arm, right: 61%	N = 44	Exp = SRG + PT Con = PT	JTT, Grip strength	4 weeks
Guo et al. (2022) [20]	RCT	China	Mean age = 57 years Mean stroke latency = 12 months Stroke type: IS (57%), HS Affected arm, right: 53%	N = 30	Exp1 = SSVEP-BCI SRG + PT + OT Exp2 = SRG + PT + OT Con = PT + OT	FMA-UE, FMA-distal UE	2, 12 weeks
Shin et al. (2022) [15]	RCT	Korea	Mean age = 60 years Mean stroke latency = 29 days Stroke type: IS (67%), HS Affected arm, right: 39%	N = 36	Exp = SRG + OT Con = OT	FMA-UE, JTT	4, 8 weeks
Wang et al. (2023) [14]	RCT	China	Mean age = 62 years Mean stroke latency = 95 days Stroke type: IS (42%), HS Affected arm, right: 49%	N = 69	Exp1 = SRG + PT + OT + Acupuncture Exp2 = rTMS + PT + OT + Acupuncture Con = PT + OT + Acupuncture	FMA-UE	2 weeks

*RCT* randomised controlled trial, *N* number, *IS* ischemic stroke, *%* percentage, *HS* haemorrhagic stroke, *Exp* experimental group, *SRG* soft robotic glove, *PT* physical therapy, *OT* occupational therapy, *Con* control group, *FMA* Fugl-Meyer Assessment scores, *UE* upper extremity, *BBT* box and blocks test score, *JTT* Jebsen–Taylor hand function test, *N/S* not stated, *SSVEP-BCI* steady-state visually evoked potentials-based brain computer interfaces, rTMS repetitive transcranial magnetic stimulation. ^a^ Forty-six participants met the inclusion criteria and underwent allocation, but only twenty-three participants completed the follow-up assessment. Intention-to-treat analysis was performed. ^b^ Twenty-three participants met the inclusion criteria and underwent allocation, but only twenty participants completed the follow-up assessment. Intention-to-treat analysis was performed.

**Table 2:** Soft robotic gloves training protocol of included studies.

Study	Type of Device	Content	Frequency (per Week)	Training Length (per Session)	Total Training Duration
Carmeli et al. (2011) [21]	HandTutor^TM^ System	Augmented wrist and fingers motion feedback Wrist flexion/extension, fingers flexion/extension Functional task training	5	20 to 30 min	3 weeks
Shin et al. (2016) [17]	RAPAEL Smart Glove	Visual biofeedback Forearm pronation/supination, wrist flexion/extension, wrist radial/ulnar deviation, finger flexion/extension Game-based functional training	5	30 min	4 weeks
Vanoglio et al. (2016) [16]	Gloreha Professional	Finger flexion/extension, thumb–finger opposition movement, wave-like finger movement	5	40 min	2 weeks
Kang et al. (2020) [19]	RAPAEL Smart Glove	Visual biofeedback Forearm pronation/supination, wrist flexion/extension, wrist radial/ulnar deviation, finger flexion/extension	5	30 min	2 weeks
Park et al. (2021) [18]	RAPAEL Smart Glove	Visual biofeedback Game-based functional training and activities of daily living	5	30 min	4 weeks
Guo et al. (2022) [20]	Soft Robotic Gloves with SSVEP-BCI or computer control	Visual biofeedback Finger flexion/extension	5	60 min ^a^	2 weeks
Shin et al. (2022) [15]	RAPAEL Smart Glove	Visual biofeedback Game-based functional training	5	30 min	4 weeks
Wang et al. (2023) [14]	Soft Robotic Glove	Wrist flexion/extension and fingers flexion/extension passively or with assistance	7	20 min	2 weeks

*SSVEP-BCI* steady-state visually evoked potentials-based brain computer interfaces. ^a^ One hour included 2 lots of 20-min trainings, a 10-min preparation at the beginning, and a 10-min rest.

**Table 3:** Subgroup analyses of the included studies.

Outcome	Categories	Studies	Participants	MD (95% CI)	Heterogeneity		*p*-Value	Egger’s Test	Quality of Evidence
					*I* ^2^	*p*-Value			
FMA-UE scores									
	All studies	**7**	222	6.52 (3.65, 9.39)	**8%**	0.37	**<0.00001**	0.98222	⊕⊕⊕◯
	Stroke latency:								
	≦six months	4	136	4.93 (0.93, 8.93)	19%	0.29	**0.02**	0.59725	⊕⊕⊕◯
	>six months	3	86	8.84 (4.47, 13.22)	0%	0.60	**<0.0001**	0.51979	⊕⊕◯◯
	Type of device:								
	RAPAEL Smart Glove	3	105	8.43 (4.27, 12.59)	0%	0.61	**<0.0001**	0.13327	⊕⊕◯◯
	Other device	4	117	5.29 (0.90, 9.67)	27%	0.25	**0.02**	0.73410	⊕⊕⊕◯
	Training length:								
	≦30 min	5	182	5.85 (2.49, 9.21)	15%	0.32	**0.0007**	0.80650	⊕⊕⊕◯
	>30 min	2	40	9.01 (2.77, 15.26)	3%	0.31	**0.005**	N/A	⊕⊕◯◯
	Total training duration:								
	≦two weeks	4	109	5.51 (0.04, 10.98)	27%	0.25	**0.05**	0.82989	⊕⊕⊕◯
	>two weeks	3	113	7.12 (3.67, 10.57)	4%	0.28	**<0.0001**	0.11801	⊕⊕◯◯
FMA-distal UE scores									
	All studies	4	109	3.27 (1.50, 5.04)	0%	0.46	**0.0003**	0.56538	⊕⊕⊕◯
	Stroke latency:								
	≦six months	1	23	−0.40 (−6.15, 5.35)	N/A	N/A	0.89	N/A	N/A
	>six months	3	86	3.66 (1.80, 5.52)	0%	0.66	**0.0001**	0.80182	⊕⊕◯◯
	Type of device:								
	RAPAEL Smart Glove	2	69	2.52 (−1.27, 6.31)	42%	0.19	0.19	N/A	⊕⊕◯◯
	Other device	2	40	3.50 (0.80, 6.20)	0%	0.37	**0.01**	N/A	⊕⊕◯◯
	Training length:								
	≦30 min	2	69	2.52 (−1.27, 6.31)	42%	0.19	0.19	N/A	⊕⊕◯◯
	>30 min	2	40	3.50 (0.80, 6.20)	0%	0.37	**0.01**	N/A	⊕⊕◯◯
	Total training duration:								
	≦two weeks	3	63	2.78 (0.15, 5.40)	11%	0.32	**0.04**	0.81777	⊕⊕◯◯
	>two weeks	1	46	3.80 (1.24, 6.36)	N/A	N/A	**0.004**	N/A	N/A
JTT scores									
	All studies	4	149	13.34 (5.16, 21.53)	8%	0.35	**0.001**	0.11011	⊕⊕⊕◯
Grip strength									
	All studies	3	94	3.11 (−6.25, 12.47)	0%	0.60	0.51	0.66567	⊕⊕◯◯
BBT scores									
	All studies	2	54	−0.75 (−9.03, 7.54)	0%	0.63	0.86	N/A	⊕⊕◯◯

*FMA* Fugl-Meyer Assessment scores, *UE* upper extremitiy, *JTT* Jebsen–Taylor hand function test, *BBT* box and blocks test score, *N/A* not assess. Bold values are significant at *p*-value < 0.05.

## Data Availability

All data relevant to this research have been extracted from the included studies. They are all included in the article or uploaded as Supplementary Materials.

## Supplementary Materials

The following supporting information can be downloaded at: https://www.mdpi.com/article/10.3390/brainsci13060900/s1; Table S1: PRISMA 2020 Checklist; Table S2: Electronic database searching strategy; Table S3: Reasons for exclusion; Table S4: Appraisal of the included studies using the GRADE tool; Figure S1: Funnel plot of studies comparing immediate and long-term FMA-UE between the soft robotic gloves and conventional rehabilitation groups; Figure S2: Funnel plot of studies comparing immediate and long-term FMA-distal UE between the soft robotic gloves and conventional rehabilitation groups; Figure S3: Mean difference (95% CI) of the immediate and long-term effect of soft robotic gloves on the Jebsen–Taylor hand function test when compared with conventional rehabilitation; Figure S4: Mean difference (95% CI) of the effect of soft robotic gloves on grip strength when compared with conventional rehabilitation; Figure S5: Mean difference (95% CI) of the effect of soft robotic gloves on box and blocks test scores when compared with conventional rehabilitation; Figure S6: Mean difference (95% CI) of baseline on FMA-UE of subacute stroke patients between groups; Figure S7: Mean difference (95% CI) of baseline on FMA-UE of chronic stroke patients between groups; and, Figure S8: Mean of the increase amount on FMA-UE of stroke patients in the subacute and chronic phases.

## Abbreviations

Fugl-Meyer assessment (FMA), Jebsen–Taylor hand function test (JTT), box and block test (BBT), soft robotic gloves (SRGs), Preferred Reporting Items for Systematic Reviews and Meta-analyses (PRISMA), randomized control trials (RCTs), post-stroke hemiparesis (PSH), conventional rehabilitation (CR), Fugl-Meyer Upper Extremity Motor Assessment (FMA-UE), Fugl-Meyer Distal Upper Extremity Motor Assessment (FMA-distal UE), standard deviation (SD), mean difference (MD), confidence intervals (CIs), Grading of Recommendations Assessment, Development and Evaluation (GRADE).
