# Supplementary material for: The Application of Soft Robotic Gloves in Stroke Patients: A Systematic Review and Meta-Analysis of Randomized Controlled Trials
Source: Brain Sci. 2023 Jun 2;13(6):900. doi: 10.3390/brainsci13060900 (PMC10295999; doi:10.3390/brainsci13060900)
Supplement: Supplementary file 1 [file brainsci-13-00900-s001.zip › brainsci-2422717-supplementary.pdf]

SUPPLEMENTARY MATERIALS

The Application of Soft Robotic Gloves in Stroke Patients: A Systematic Review and Meta-analysis of Randomized Controlled Trials

Contents

Supplementary Material S1 -PRISMA 2020 Checklist.

Supplementary Material S2 - Electronic database searching strategy.

Supplementary Material S3 - Reasons for exclusion (n=148).

Supplementary Material S4 - Appraisal of the included studies using the GRADE tool.

Supplementary Material S5 - Funnel plots.

Supplementary Material S6 - Forest plots of secondary outcomes

Supplementary Material S7 - Forest plots of FMA-UE baseline

Supplementary Material S1 - Table S1. PRISMA 2020 Checklist.

Section and Topic	Item #	Checklist item	Location where item is reported
**TITLE**			
Title	1	Identify the report as a systematic review.	1
**ABSTRACT**			
Abstract	2	Follow the guideline of PRISMA 2020 for Abstracts checklist.	1
**INTRODUCTION**			
Rationale	3	Describe the rationale for the review in the context of existing knowledge.	2
Objectives	4	Provide an explicit statement of the objective(s) or question(s) the review addresses.	2
**METHODS**			
Eligibility criteria	5	Specify the inclusion and exclusion criteria for the review and how studies were grouped for the syntheses.	2,3
Information sources	6	Specify all databases, registers, websites, organisations, reference lists and other sources searched or consulted to identify studies. Specify the date when each source was last searched or consulted.	2,3
Search strategy	7	Present the full search strategies for all databases, registers and websites, including any filters and limits used.	2,3
Selection process	8	Specify the methods used to decide whether a study met the inclusion criteria of the review, including how many	2,3

**Section and Topic**	**Item #**	**Checklist item**	**Location where item is reported**
		reviewers screened each record and each report retrieved, whether they worked independently, and if applicable, details of automation tools used in the process.	
Data collection process	9	Specify the methods used to collect data from reports, including how many reviewers collected data from each report, whether they worked independently, any processes for obtaining or confirming data from study investigators, and if applicable, details of automation tools used in the process.	3
Data items	10a	List and define all outcomes for which data were sought. Specify whether all results that were compatible with each outcome domain in each study were sought (e.g. for all measures, time points, analyses), and if not, the methods used to decide which results to collect.	3
	10b	List and define all other variables for which data were sought (e.g. participant and intervention characteristics, funding sources). Describe any assumptions made about any missing or unclear information.	3
Study risk of bias assessment	11	Specify the methods used to assess risk of bias in the included studies, including details of the tool(s) used, how many reviewers assessed each study and whether they worked independently, and if applicable, details of automation tools used in the process.	3
Effect measures	12	Specify for each outcome the effect measure(s) (e.g. risk ratio, mean difference) used in the synthesis or presentation of	3

Section and Topic	Item #	Checklist item	Location where item is reported
		results.	
Synthesis methods	13a	Describe the processes used to decide which studies were eligible for each synthesis (e.g. tabulating the study intervention characteristics and comparing against the planned groups for each synthesis (item #5)).	3
	13b	Describe any methods required to prepare the data for presentation or synthesis, such as handling of missing summary statistics, or data conversions.	3
	13c	Describe any methods used to tabulate or visually display results of individual studies and syntheses.	3
	13d	Describe any methods used to synthesize results and provide a rationale for the choice(s). If meta-analysis was performed, describe the model(s), method(s) to identify the presence and extent of statistical heterogeneity, and software package(s) used.	3
	13e	Describe any methods used to explore possible causes of heterogeneity among study results (e.g. subgroup analysis, meta-regression).	3
	13f	Describe any sensitivity analyses conducted to assess robustness of the synthesized results.	3
Reporting bias assessment	14	Describe any methods used to assess risk of bias due to missing results in a synthesis (arising from reporting biases).	3

Section and Topic	Item #	Checklist item	Location where item is reported
Certainty assessment	15	Describe any methods used to assess certainty (or confidence) in the body of evidence for an outcome.	3
**RESULTS**			
Study selection	16a	Describe the results of the search and selection process, from the number of records identified in the search to the number of studies included in the review, ideally using a flow diagram.	4, Figure 1
	16b	Cite studies that might appear to meet the inclusion criteria, but which were excluded, and explain why they were excluded.	4
Study characteristics	17	Cite each included study and present its characteristics.	4, 5, 6, Table 1
Risk of bias in studies	18	Present assessments of risk of bias for each included study.	8, Figure 2
Results of individual studies	19	For all outcomes, present, for each study: (a) summary statistics for each group (where appropriate) and (b) an effect estimate and its precision (e.g. confidence/credible interval), ideally using structured tables or plots.	8, 9, Figure 3,4,
Results of	20a	For each synthesis, briefly summarise the characteristics and risk of bias among contributing studies.	8, 9, 12

Section and Topic	Item #	Checklist item	Location where item is reported
syntheses	20b	Present results of all statistical syntheses conducted. If meta-analysis was done, present for each the summary estimate and its precision (e.g. confidence/credible interval) and measures of statistical heterogeneity. If comparing groups, describe the direction of the effect.	8, 9, 12
	20c	Present results of all investigations of possible causes of heterogeneity among study results.	10, 11, 12, Table 3
	20d	Present results of all sensitivity analyses conducted to assess the robustness of the synthesized results.	8, 9, 12
Reporting biases	21	Present assessments of risk of bias due to missing results (arising from reporting biases) for each synthesis assessed.	8, Figure 2
Certainty of evidence	22	Present assessments of certainty (or confidence) in the body of evidence for each outcome assessed.	Table S4
**DISCUSSION**			
Discussion	23a	Provide a general interpretation of the results in the context of other evidence.	12, 13, 14
	23b	Discuss any limitations of the evidence included in the review.	14
	23c	Discuss any limitations of the review processes used.	14
	23d	Discuss implications of the results for practice, policy, and future research.	14
**OTHER INFORMATION**			

Section and Topic	Item #	Checklist item	Location where item is reported
Registration and protocol	24a	Provide registration information for the review, including register name and registration number, or state that the review was not registered.	2, 3
	24b	Indicate where the review protocol can be accessed, or state that a protocol was not prepared.	2, 3
	24c	Describe and explain any amendments to information provided at registration or in the protocol.	2, 3
Support	25	Describe sources of financial or non-financial support for the review, and the role of the funders or sponsors in the review.	15
Competing interests	26	Declare any competing interests of review authors.	15
Availability of data, code and other materials	27	Report which of the following are publicly available and where they can be found: template data collection forms; data extracted from included studies; data used for all analyses; analytic code; any other materials used in the review.	15

14 OR, online resource.

Supplementary Material S2 - Table S2. Electronic database searching strategy.

**Electronic search strategy**		
**PubMed search**		
Population/Stroke	#1	("Soft robotic glove" OR "Soft wearable robot" OR "Soft Robotic" OR "Robotic glove" OR "Robotic, Soft" OR "Wearable robotic" OR "Glove")
Intervention/Soft robotic glove	#2	("Stroke"[Mesh] OR "Stroke, Lacunar"[Mesh] OR "National Institute of Neurological Disorders and Stroke (U.S.)"[Mesh] OR "Hemorrhagic Stroke"[Mesh] OR "Embolic Stroke"[Mesh] OR "Thrombotic Stroke"[Mesh] OR "Ischemic Stroke"[Mesh] OR "Stroke Rehabilitation"[Mesh] OR "Infarction, Posterior Cerebral Artery"[Mesh] OR "Brain Stem Infarctions"[Mesh] OR "Infarction, Middle Cerebral Artery"[Mesh] OR "Infarction, Anterior Cerebral Artery"[Mesh] OR "Anterior spinal artery stroke")
Filters		None
Search algorithm	#3	#1 AND #2
**Cochrane Library Databases search** (Title, Abstract, Keywords)		
Population/Stroke	#1	("Stroke" OR "Hemorrhagic Stroke" OR "Embolic Stroke" OR "Thrombotic Stroke" OR "Ischemic Stroke")
Intervention/Soft robotic glove	#2	("Soft robotic glove" OR "Soft wearable robot" OR "Soft Robotic" OR "Robotic glove" OR "Robotic, Soft" OR "Wearable robotic" OR "Glove")
Filters		None
Search algorithm	#3	#1 AND #2
**Embase search** (Title, Abstract, Author keywords)		
Population/Stroke	#1	("Stroke" OR "Hemorrhagic Stroke" OR "Embolic Stroke" OR "Thrombotic Stroke" OR "Ischemic Stroke" OR "Spinal Cord Injuries")

Intervention/Soft robotic glove	#2	("Soft robotic glove" OR "Soft wearable robot" OR "Soft Robotic" OR "Robotic glove" OR "Robotic, Soft" OR "Wearable robotic" OR "Glove")
Filters	#3	None
Search algorithm	#4	#1 AND #2
**Web of Science search**		
Population/Stroke	#1	("Stroke" OR "Hemorrhagic Stroke" OR "Embolic Stroke" OR "Thrombotic Stroke" OR "Ischemic Stroke" OR "Spinal Cord Injuries")
Intervention/Soft robotic glove	#2	("Soft robotic glove" OR "Soft wearable robot" OR "Soft Robotic" OR "Robotic glove" OR "Robotic, Soft" OR "Wearable robotic" OR "Glove")
Filters		None
Search algorithm	#3	#1 AND #2
**Embase**		
Population/Stroke	#1	("Soft robotic glove" OR "Soft wearable robot" OR "Soft Robotic" OR "Robotic glove" OR "Robotic, Soft" OR "Wearable robotic" OR "Glove")
Intervention/Soft robotic glove	#2	("Stroke" OR "Hemorrhagic Stroke" OR "Embolic Stroke" OR "Thrombotic Stroke" OR "Ischemic Stroke")
Filters		None
Search algorithm	#3	#1 AND #2
**PEDro search**		
Abstract and Title	#1	("Soft robotic glove" OR "Soft wearable robot" OR "Soft Robotic" OR "Robotic glove" OR "Robotic, Soft" OR "Wearable robotic" OR "Glove")
Problem	#2	None
Subdiscipline	#3	None
Method	#4	None
Search algorithm	#5	#1

Supplementary Material S3 - Table S3. Reasons for exclusion (n=148).

Study		Title of the article	Additional notes
1.	Luo 2020	Synergistic Effect of Combined Mirror Therapy on Upper Extremity in Patients With Stroke: A Systematic Review and Meta-Analysis.	Review article was excluded
2.	Fardipour 2022	Investigation of therapeutic effects of wearable robotic gloves on improving hand function in stroke patients- A systematic review	Review article was excluded
3.	Fernandez 2022	Haptic Glove Systems in Combination with Semi-Immersive Virtual Reality for Upper Extremity Motor Rehabilitation after Stroke: A Systematic Review and Meta-Analysis	Review article was excluded
4.	Kabir 2022	Hand Rehabilitation Devices: A Comprehensive Systematic Review	Review article was excluded
5.	Dávila-Vilchis 2022	Design Criteria of Soft Exogloves for Hand Rehabilitation Assistance Tasks	Review article was excluded
6.	Liu 2022	Current State of Robotics in Hand Rehabilitation after Stroke: A Systematic Review	Review article was excluded
7.	Veale 2016	Towards compliant and wearable robotic orthoses: A review of current and emerging actuator technologies	Review article was excluded
8.	Hernández 2023	Hand robotic devices in neurorehabilitation: A systematic review on the feasibility and effectiveness of stroke rehabilitation	Review article was excluded
9.	Connelly 2010	A Pneumatic Glove and Immersive Virtual Reality Environment for Hand Rehabilitative Training After Stroke	Cohort study was excluded
10.	Thielbar 2017	Benefits of using a voice and EMG-driven actuated glove to support occupational therapy for stroke survivors	Cohort study was excluded
11.	Qiu 2022	Synergistic Immediate Cortical Activation on Mirror Visual Feedback Combined With a Soft	Not met our **P**ICO(Participants were not stroke patients)

		Robotic Bilateral Hand Rehabilitation System: A Functional Near Infrared Spectroscopy Study	
12.	Lin 2016	Development of a novel haptic glove for improving finger dexterity in poststroke rehabilitation	Not met our **P**ICO(Participants were healthy people)
13.	Ku 2003	A data glove with tactile feedback for fMRI of virtual reality experiments	Not met our **P**ICO(Participants were healthy people)
14.	Krammer 2020	Sensing form - finger gaiting as key to tactile object exploration - a data glove analysis of a prototypical daily task	Not met our **P**ICO(Participants were healthy people)
15.	Jha 2021	Design and Evaluation of an FBG Sensor-Based Glove to Simultaneously Monitor Flexure of Ten Finger Joints.	Not met our **P**ICO(Participants were healthy people)
16.	Oess 2012	Design and evaluation of a low-cost instrumented glove for hand function assessment	Not met our **P**ICO(Participants were patients with cervical spinal cord injury)
17.	Zondervan 2016	Home-based hand rehabilitation after chronic stroke: Randomized, controlled single-blind trial comparing the MusicGlove with a conventional exercise program	Not met our P**I**CO(therapy with music glove may confound the effect of soft robotic glove)
18.	Sanders 2020	easibility of Wearable Sensing for In-Home Finger Rehabilitation Early After Stroke	Not met our P**I**CO(therapy with music glove may confound the effect of soft robotic glove)
19.	Friedman 2014	Retraining and assessing hand movement after stroke using the MusicGlove: comparison with conventional hand therapy and isometric grip training	Not met our P**I**CO(therapy with music glove may confound the effect of soft robotic glove)
20.	Friedman 2011	MusicGlove: Motivating and Quantifying Hand Movement Rehabilitation by using Functional Grips to Play Music	Not met our P**I**CO(therapy with music glove may confound the effect of soft robotic glove)
21.	Hoda 2015	A Novel Study on Natural Robotic Rehabilitation Exergames Using the Unaffected Arm of Stroke Patients	Not met our P**I**CO(The rehabilitation glove was worn on the unaffected arm)

22.	Krukowska 2014	Influence of the surface electrostimulation controlled by muscle contraction on the bioelectric muscle activity and restoration of the hand function in cerebral stroke patients	Not met our P**I**CO (Focus on the effect of surface electrostimulation)
23.	Sullivan 2019	Afferent stimulation provided by glove electrode during task-specific arm exercise following stroke	Not met our P**I**CO (Focus on the effect of sensory amplitude electrical stimulation)
24.	Cheng 2020	Brain-Computer Interface-Based Soft Robotic Glove Rehabilitation for Stroke	Not met our P**I**CO (Focus on the effect of intervention with brain-computer)
25.	Kim 2022	Increasing motor cortex activation during grasping via novel robotic mirror hand therapy: a pilot fNIRS study	Not met our P**I**CO (Evaluation of the theraputic effect of mirror therapy)
26.	Almarzouki 2021	An Internet of Medical Things-Based Model for Real-Time Monitoring and Averting Stroke Sensors	Not met our P**I**CO(Not using glove for rehabilitation)
27.	Dutta 2022	Poststroke Grasp Ability Assessment Using an Intelligent Data Glove Based on Action Research Arm Test Development, Algorithms, and Experiments	Not met our P**I**CO(The glove was used for assessment of grasp ability)
28.	Henderson 2012	Reliability and Validity of Clinically Accessible Smart Glove Technologies to Measure Joint Range of Motion	Not met our P**I**CO(The glove was used for measuring joint range of motion)
29.	Vélez-Guerrero 2022	Assessment of the Mechanical Support Characteristics of a Light and Wearable Robotic Exoskeleton Prototype Applied to Upper Limb Rehabilitation	Not met our P**I**CO (Not soft robotic glove)
30.	Vélez-Guerrero 2021	Design, Development, and Testing of an Intelligent Wearable Robotic Exoskeleton Prototype for Upper Limb Rehabilitation	Not met our P**I**CO (Not soft robotic glove)
31.	Ooi 2020	Effects of pressure garment on spasticity and function of the arm in the early stages after stroke: a randomized controlled trial	Not met our P**I**CO (Not soft robotic glove)
32.	Yang 2021	Biomechanical Evaluation of Dynamic Splint	Not met our P**I**CO (Not soft robotic

		Based on Pulley Rotation Design for Management of Hand Spasticity	glove)
33.	Casas 2021	Clinical Test of a Wearable, High DOF, Spring Powered Hand Exoskeleton (HandSOME II)	Not met our P**I**CO (Not soft robotic glove)
34.	Triandafilou 2014	Carryover effects of cyclical stretching of the digits on hand function in stroke survivors	Not met our P**I**CO (Not soft robotic glove)
35.	Schrader 2022	The effect of mirror therapy can be improved by simultaneous robotic assistance	Not met our P**I**CO (Not soft robotic glove)
36.	Sarac 2019	Design Requirements of Generic Hand Exoskeletons and Survey of Hand Exoskeletons for Rehabilitation, Assistive, or Haptic Use	Not met our P**I**CO (Not soft robotic glove)
37.	Sandison 2020	HandMATE_ Wearable Robotic Hand Exoskeleton and Integrated Android App for At Home Stroke Rehabilitation	Not met our P**I**CO (Not soft robotic glove)
38.	Salvietti 2017	Compensating Hand Function in Chronic Stroke Patients Through the Robotic Sixth Finger	Not met our P**I**CO (Not soft robotic glove)
39.	Vanteddu 2020	Stable Grasp Control With a Robotic Exoskeleton Glove	Not met our P**I**CO (Not soft robotic glove)
40.	Ranzani 2020	Neurocognitive robot-assisted rehabilitation of hand function: a randomized control trial on motor recovery in subacute stroke	Not met our P**I**CO (Not soft robotic glove)
41.	Pu 2020	Robotic hand system design for mirror therapy rehabilitation after stroke	Not met our P**I**CO (Not soft robotic glove)
42.	Park 2019	Multimodal Sensing and Interaction for a Robotic Hand Orthosis	Not met our P**I**CO (Not soft robotic glove)
43.	Li 2019	An Attention-Controlled Hand Exoskeleton for the Rehabilitation of Finger Extension and Flexion Using a Rigid-Soft Combined Mechanism	Not met our P**I**CO (Not soft robotic glove)
44.	Kim 2013	Kinematic Data Analysis for Post-Stroke Patients Following Bilateral Versus Unilateral Rehabilitation With an Upper Limb Wearable Robotic System	Not met our P**I**CO (Not soft robotic glove)
45.	Gilmartin 2017	A robotic hand exoskeleton for rehabilitation	Not met our P**I**CO (Not soft robotic

		following stroke	glove)
46.	Friedman 2014	The Manumeter: A Wearable Device for Monitoring Daily Use of the Wrist and Fingers	Not met our P**I**CO (Not soft robotic glove)
47.	Fischer 2016	Use of a Portable Assistive Glove to Facilitate Rehabilitation in Stroke Survivors With Severe Hand Impairment	Not met our P**I**CO (Not soft robotic glove)
48.	Cordella 2020	Hand motion analysis during robot-aided rehabilitation in chronic stroke	Not met our P**I**CO (Not soft robotic glove)
49.	Cheng 2018	Design and Control of a Wearable Hand Rehabilitation Robot	Not met our P**I**CO (Not soft robotic glove)
50.	Chen 2020	Development of a Novel Task-oriented Rehabilitation Program using a Bimanual Exoskeleton Robotic Hand	Not met our P**I**CO (Not soft robotic glove)
51.	Lin 2012	Effect of mirror therapy combined with somatosensory stimulation on motor recovery and daily function in stroke patients	Not met our P**I**CO (Not soft robotic glove)
52.	Lee 2015	Combining Afferent Stimulation and Mirror Therapy for Improving Muscular, Sensorimotor, and Daily Functions After Chronic Stroke	Not met our P**I**CO (Not soft robotic glove)
53.	Trompetto 2022	A soft supernumerary hand for rehabilitation in sub-acute stroke: a pilot study	Not met our P**I**CO (Not soft robotic glove)
54.	Prange 2017	Applying a soft-robotic glove as assistive device and training tool with games to support hand function after stroke: preliminary results on feasibility and potential clinical impact	Not met our PI**C**O(No control group)
55.	Wu 2017	The potential effect of a vibrotactile glove rehabilitation system on motor recovery in chronic post-stroke hemiparesis	Not met our PI**C**O(No control group)
56.	Shi 2021	Effects of a Soft Robotic Hand for Hand Rehabilitation in Chronic Stroke Survivors	Not met our PI**C**O(No control group)
57.	Lansberg 2022	Home-based virtual reality therapy for hand recovery after stroke	Not met our PI**C**O(No control group)

58.	Connelly 2009	Use of a pneumatic glove for hand rehabilitation following stroke	Not met our PICO(No control group)
59.	Ciullo 2020	A Novel Soft Robotic Supernumerary Hand for Severely Affected Stroke Patients	Not met our PICO(No control group)
60.	Bernocchi 2018	Home-based hand rehabilitation with a robotic glove in hemiplegic patients after stroke: a pilot feasibility study	Not met our PICO(No control group)
61.	SEIM 2021	Wearable vibrotactile stimulation for upper extremity rehabilitation in chronic stroke: clinical feasibility trial using the VTS Glove	Not met our PICO(No control group)
62.	Heung 2019	Robotic Glove with Soft-Elastic Composite Actuators for Assisting Activities of Daily Living	Not met our PICO(No control group)
63.	Kim 2020	A Wearable Soft Robot for Stroke Patients' Finger Occupational Therapy and Quantitative Measures on the Joint Paralysis	Not met our PICO(No control group)
64.	Yurkewich 2020	Hand Extension Robot Orthosis (HERO) Grip Glove: enabling independence amongst persons with severe hand impairments after stroke	Not met our PICO(No control group)
65.	Song 2022	Proposal of a Wearable Multimodal Sensing-Based Serious Games Approach for Hand Movement Training After Stroke	Not met our PICO(No control group)
66.	Jeong 2022	Soft Wearable Robot With Shape Memory Alloy (SMA)-Based Artificial Muscle for Assisting With Elbow Flexion and Forearm Supination/Pronation	Not met our PICO(No control group)
67.	Hussain 2017	A soft supernumerary robotic finger and mobile arm support for grasping compensation and hemiparetic upper limb rehabilitation	Not met our PICO(No control group)
68.	Haghshenas-Jaryani 2020	A pilot study on the design and validation of a hybrid exoskeleton robotic device for hand rehabilitation	Not met our PICO(No control group)
69.	Ranganathan 2017	Reorganization of finger coordination patterns through motor exploration in	Not met our PICO(No control group)

		individuals after stroke	
70.	Radder 2018	Feasibility of a wearable soft-robotic glove to support impaired hand function in stroke patients	Not met our PI**C**O(No control group)
71.	Lin 2017	Data glove system embedded with inertial measurement units for hand function evaluation in stroke patients	Not met our PI**C**O(No control group)
72.	Ryser 2017	Fully Embedded Myoelectric Control for a Wearable Robotic Hand Orthosis	Not met our PI**C**O(No control group)
73.	Wang 2019	Design and Testing of a Soft Rehabilitation Glove Integrating Finger and Wrist Function.	Not met our PI**C**O(No control group)
74.	Yap 2017	Design and Preliminary Feasibility Study of a Soft Robotic Glove for Hand Function Assistance in Stroke Survivors	Not met our PI**C**O(No control group)
75.	Sullivan 2015	Improving arm function in chronic stroke: a pilot study of sensory amplitude electrical stimulation via glove electrode during task-specific training	Not met our PIC**O**(Evaluation of the effect on arm instead of hand)
76.	Nasrallah 2021	Effect of proprioceptive stimulation using a soft robotic glove on motor activation and brain connectivity in stroke survivors	Not met our PIC**O** (Evaluation of the effect on brain networks)
77.	Leem 2019	Predictors of functional and motor outcomes following upper limb robot-assisted therapy after stroke	Not met our PIC**O** (Evaluation of the ideal predictors of functional and motor outcomes)
78.	Adams 2019	Upper Extremity Function Assessment Using a Glove Orthosis and Virtual Reality System	Not met our PIC**O** (Desired outcome not reported)
79.	Dutta 2016	Bayesian network aided grasp and grip efficiency estimation using a smart data glove for post-stroke diagnosis	Not met our PIC**O** (Desired outcome not reported)
80.	Nathan 2009	Design and validation of low-cost assistive glove for hand assessment and therapy during activity of daily living-focused robotic stroke therapy	Not met our PIC**O** (Desired outcome not reported)
81.	Alnajjar 2021	CHAD: Compact Hand-Assistive Device for	Not met our PIC**O** (Desired outcome

		enhancement of function in hand impairments	not reported)
82.	Tunik 2013	Visuomotor discordance during visually-guided hand movement in virtual reality modulates sensorimotor cortical activity in healthy and hemiparetic subjects	Not met our PIC**O** (Desired outcome not reported)
83.	Fei 2021	Development of a Wearable Glove System with Multiple Sensors for Hand Kinematics Assessment	Not met our PIC**O** (Desired outcome not reported)
84.	Tang 2022	Probabilistic Model-Based Learning Control of a Soft Pneumatic Glove for Hand Rehabilitation	Not met our PIC**O** (Desired outcome not reported)
85.	Tang 2021	Model-based online learning and adaptive control for a “human-wearable soft robot” integrated system	Not met our PIC**O** (Desired outcome not reported)
86.	Lin 2019	A Modular Data Glove System for Finger and Hand Motion Capture Based on Inertial Sensors	Not met our PIC**O** (Desired outcome not reported)
87.	Li 2017	E Glove Evaluation & Training System Based on ARAT and Fusion of Visual and Tactile Information	Not met our PIC**O** (Desired outcome not reported)
88.	Gerez 2020	A Hybrid, Wearable Exoskeleton Glove Equipped With Variable Stiffness Joints, Abduction Capabilities, and a Telescopic Thumb	Not met our PIC**O** (Desired outcome not reported)
89.	O'Neill 2020	Inflatable Soft Wearable Robot for Reducing Therapist Fatigue During Upper Extremity Rehabilitation in Severe Stroke	Not met our PIC**O** (Desired outcome not reported)
90.	McCall 2021	High Compliance Pneumatic Actuators to Promote Finger Extension in Stroke Survivors	Not met our PIC**O** (Desired outcome not reported)
91.	Palmcrantz 2020	Factors affecting the usability of an assistive soft robotic glove after stroke or multiple sclerosis	Not met our PIC**O** (Desired outcome not reported)
92.	Varalta 2014	Effects of contralesional robot-assisted hand-training in patients with unilateral spatial neglect following stroke	Case serials were excluded
93.	Heung 2019	Design of a 3D Printed Soft Robotic Hand for Stroke Rehabilitation and Daily Activities	Case serials were excluded

		Assistance	
94.	Boian 2002	Virtual reality-based post-stroke hand rehabilitation	Case serials were excluded
95.	Adamovich 2003	A virtual reality based exercise system for hand rehabilitation post-stroke: transfer to function	Case serials were excluded
96.	Rieger 2022	A Preliminary Study to Design and Evaluate Pneumatically Controlled Soft Robotic Actuators for a Repetitive Hand Rehabilitation Task	Case serials were excluded
97.	Colovic 2020	Upper limb robotic neurorehabilitation after pediatric stroke	Case report was excluded
98.	Nuckols 2019	Proof of Concept of Soft Robotic Glove for Hand Rehabilitation in Stroke Survivors	Poster was excluded
99.	Koenig 2013	Modulation of cortical plasticity by whole-hand electrical stimulation in attempt to improve hand motor functions after stroke	Poster was excluded
100.	Kim 2018	Effects of digital smart glove system on motor recovery of upper extremity in subacute stroke patients	Poster was excluded
101.	Vorokhta 2021	Recovery of motor function of the upper limbs after a stroke	Poster was excluded
102.	Proulx 2020	Perceived Usability and Acceptability of a Soft Robotic Glove for Rehabilitation of Adults With Hand Hemiparesis: A Mixed-Method Study Among Occupational Therapists in Stroke Rehabilitation	Poster was excluded
103.	Bhasin 2022	Development and Design of Piezoelectric Hand Glove for Upper Limb Hunction post Stroke	Poster was excluded
104.	Albart 2021	The comparison of robotic glove training and conventional training in post-stroke hand impairment patients: A pilot randomized controlled trial protocol	Pilot randomized controlled trial protocol was excluded
105.	Alexander 2021	SaeboGlove therapy for upper limb disability and severe hand impairment	Study protocol without outcome was excluded

		after stroke (SUSHI): Study protocol for a randomised controlled trial	
106.	Proietti 2021	Sensing and Control of a Multi-Joint Soft Wearable Robot for Upper-Limb Assistance and Rehabilitation	Not clinical trial
107.	Yap 2017	A Fully Fabric-Based Bidirectional Soft Robotic Glove for Assistance and Rehabilitation of Hand Impaired Patients	Not clinical trial
108.	Yap 2016	Design of a Soft Robotic Glove for Hand Rehabilitation of Stroke Patients With Clenched Fist Deformity Using Inflatable Plastic Actuators	Not clinical trial
109.	Gerez 2019	On the Development of Adaptive, Tendon-Driven, Wearable Exo-Gloves for Grasping Capabilities Enhancement	Not clinical trial
110.	Van 2019	Detection of the Intention to Grasp During Reaching in Stroke Using Inertial Sensing	Not clinical trial
111.	Kim 2021	Slider-Tendon Linear Actuator with Under-actuation and Fast-connection for Soft Wearable Robots	Not clinical trial
112.	Choi 2019	Exo-Wrist: A Soft Tendon-Driven Wrist-Wearable Robot with Active Anchor for Dart-Throwing Motion in Hemiplegic Patients	Not clinical trial
113.	Wu 2022	A Twisted and Coiled Polymer Artificial Muscles Driven Soft Crawling Robot Based on Enhanced Antagonistic Configuration	Not clinical trial
114.	Lee 2022	Recent-advances-in-wearable-exoskeletons-for-human-strength-augmentation	Not clinical trial
115.	Yang 2021	An Instrumented Glove-Controlled Portable Hand-Exoskeleton for Bilateral Hand Rehabilitation	Not clinical trial
116.	Zhao 2020	Wearable Physiological Monitoring System Based on Electrocardiography and Electromyography for Upper Limb Rehabilitation Training	Not clinical trial

117.	Chen 2021	A Wearable Hand Rehabilitation System With Soft Gloves	Not clinical trial
118.	Carbonaro 2014	Exploiting wearable goniometer technology for motion sensing gloves	Not clinical trial
119.	Biggar 2016	Design and Evaluation of a Soft and Wearable Robotic Glove for Hand Rehabilitation	Not clinical trial
120.	Haghshenas-Jaryani 2019	Soft Robotic Bilateral Hand Rehabilitation System for Fine Motor Learning	Not clinical trial
121.	Han 2022	Design of Wearable Hand Rehabilitation Glove With Bionic Fiber-Reinforced Actuator	Not clinical trial
122.	Proulx 2021	Occupational therapists' evaluation of the perceived usability and utility of wearable soft robotic exoskeleton gloves for hand function rehabilitation following a stroke	Not clinical trial
123.	Placidi 2018	Measurements by A LEAP-Based Virtual Glove for the Hand Rehabilitation	Not clinical trial
124.	Coffey 2014	A Novel BCI-Controlled Pneumatic Glove System for Home-Based Neurorehabilitation	Not clinical trial
125.	Ochoa 2009	Development of a portable actuated orthotic glove to facilitate gross extension of the digits for therapeutic training after stroke	Not clinical trial
126.	Jumphoo 2021	Soft Robotic Glove Controlling Using Brainwave Detection for Continuous Rehabilitation at Home	Not clinical trial
127.	Rudd 2019	A Low-Cost Soft Robotic Hand Exoskeleton for Use in Therapy of Limited Hand–Motor Function	Not clinical trial
128.	Kim 2020	Joint Angle Estimation of a Tendon-Driven Soft Wearable Robot through a Tension and Stroke Measurement	Not clinical trial
129.	Kim 2019	Development of an interactive game-based mirror image hand rehabilitation system	Not clinical trial

130.	Seim 2022	Design of a Wearable Vibrotactile Stimulation Device for Individuals With Upper-Limb Hemiparesis and Spasticity	Not clinical trial
131.	Polygerinos 2015	Soft robotic glove for combined assistance and at-home rehabilitation	Not clinical trial
132.	Park 2020	A Finger Grip Force Sensor with an Open-Pad Structure for Glove-Type Assistive Devices	Not clinical trial
133.	Lee 2019	Long Shape Memory Alloy Tendon-based Soft Robotic Actuators and Implementation as a Soft Gripper	Not clinical trial
134.	Jha 2019	An FBG-Based Sensing Glove to Measure Dynamic Finger Flexure With an Angular Resolution of 0.1◦ up to Speeds of 80◦/s	Not clinical trial
135.	Jeong 2019	Design of Shape Memory Alloy-Based Soft Wearable Robot for Assisting Wrist Motion	Not clinical trial
136.	Burns 2021	Design and Implementation of an Instrumented Data Glove that measures Kinematics and Dynamics of Human Hand	Not clinical trial
137.	Ayodele 2021	A Weft Knit Data Glove	Not clinical trial
138.	Ahmed 2021	Flexohand: A Hybrid Exoskeleton-Based Novel Hand Rehabilitation Device	Not clinical trial
139.	Ahmadjou 2021	A compact valveless pressure control source for soft rehabilitation glove	Not clinical trial
140.	Chen 2022	Soft Exoskeleton With Fully Actuated Thumb Movements for Grasping Assistance	Not clinical trial
141.	Placidi 2013	Overall design and implementation of the virtual glove	Not clinical trial
142.	Wang 2022	A soft pneumatic glove with multiple rehabilitation postures and assisted grasping modes	Not clinical trial
143.	Park 2023	Portable 3D-printed hand orthosis with spatial stiffness distribution personalized for assisting	Not clinical trial

		grasping in daily living	
144.	Han 2022	Design of Wearable Hand Rehabilitation Glove With Bionic Fiber-Reinforced Actuator	Not clinical trial
145.	Kim 2022	Bioinspired High-Degrees of Freedom Soft Robotic Glove for Restoring Versatile and Comfortable Manipulation	Not clinical trial
146.	Guo 2023	Kirigami-Inspired 3D Printable Soft Pneumatic Actuators with Multiple Deformation Modes for Soft Robotic Applications	Not clinical trial
147.	Thimabut 2022	Effectiveness of a Soft Robotic Glove to Assist Hand Function in Stroke Patients: A Cross-Sectional Pilot Study	The baseline information of two groups were not clear
148.	Grigoras 2016	Testing of a Hybrid FES-Robot Assisted Hand Motor Training Program in Sub-Acute Stroke Survivors	Incomplete data

P, patient; I. intervention; C, comparison; O, outcome.

Supplementary Material S4 - Table S4. Appraisal of the included studies using the GRADE tool.

Quality assessment							Quality of evidence
No. of studies	Design	Risk of bias	Inconsistency	Indirectness	Imprecision	Publication bias	
			FMA-UE scores (Soft robotic gloves versus conventional rehabilitation)				
7 (222 patients)	RCT	No serious risk of bias	No serious inconsistency	No serious indirectness	Serious[a]	No serious publication bias	⊕⊕⊕○ Moderate
			FMA-distal UE scores (Soft robotic gloves versus conventional rehabilitation)				
3 (109 patients)	RCT	No serious risk of bias	No serious inconsistency	No serious indirectness	Serious[a]	No serious publication bias	⊕⊕⊕○ Moderate
			JTT scores (Soft robotic gloves versus conventional rehabilitation)				
4 (149 patients)	RCT	No serious risk of bias	No serious inconsistency	No serious indirectness	Serious[a]	No serious publication bias	⊕⊕⊕○ Moderate
			Grip strength (Soft robotic gloves versus conventional rehabilitation)				
3 (94 patients)	RCT	No serious risk of bias	No serious inconsistency	No serious indirectness	Serious[a,b]	Serious[c]	⊕⊕○○ Low
			BBT scores (Soft robotic gloves versus conventional rehabilitation)				
2 (54 patients)	RCT	No serious risk of bias	No serious inconsistency	No serious indirectness	Serious[a,b]	Serious[d]	⊕⊕○○ Low

No., number; FMA, Fugl-Meyer Assessment scores; UE, upper extremitiy; RCT, randomized control trial; JTT, Jebsen–Taylor hand function test; BBT box and blocks test score

[a] Studies contained a small sample size.

[b] 95% confidence interval met median line.

[c] Only three studies met the criteria for analysis.

[d] Only two studies met the criteria for analysis.

Supplementary Material S5. - Funnel plots.

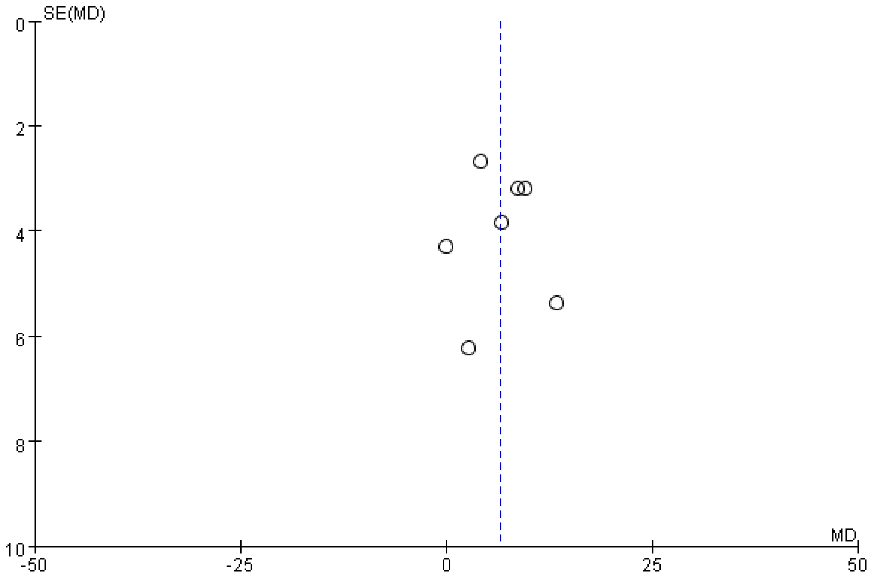

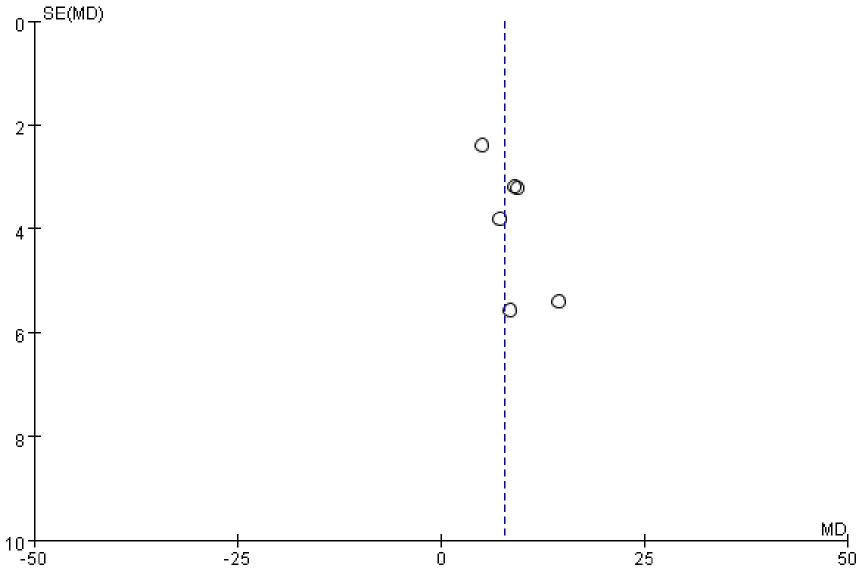

Figure S1. Funnel plot of studies comparing (a) immediate and (b) long-term FMA-UE between the soft robotic gloves and conventional rehabilitation groups.

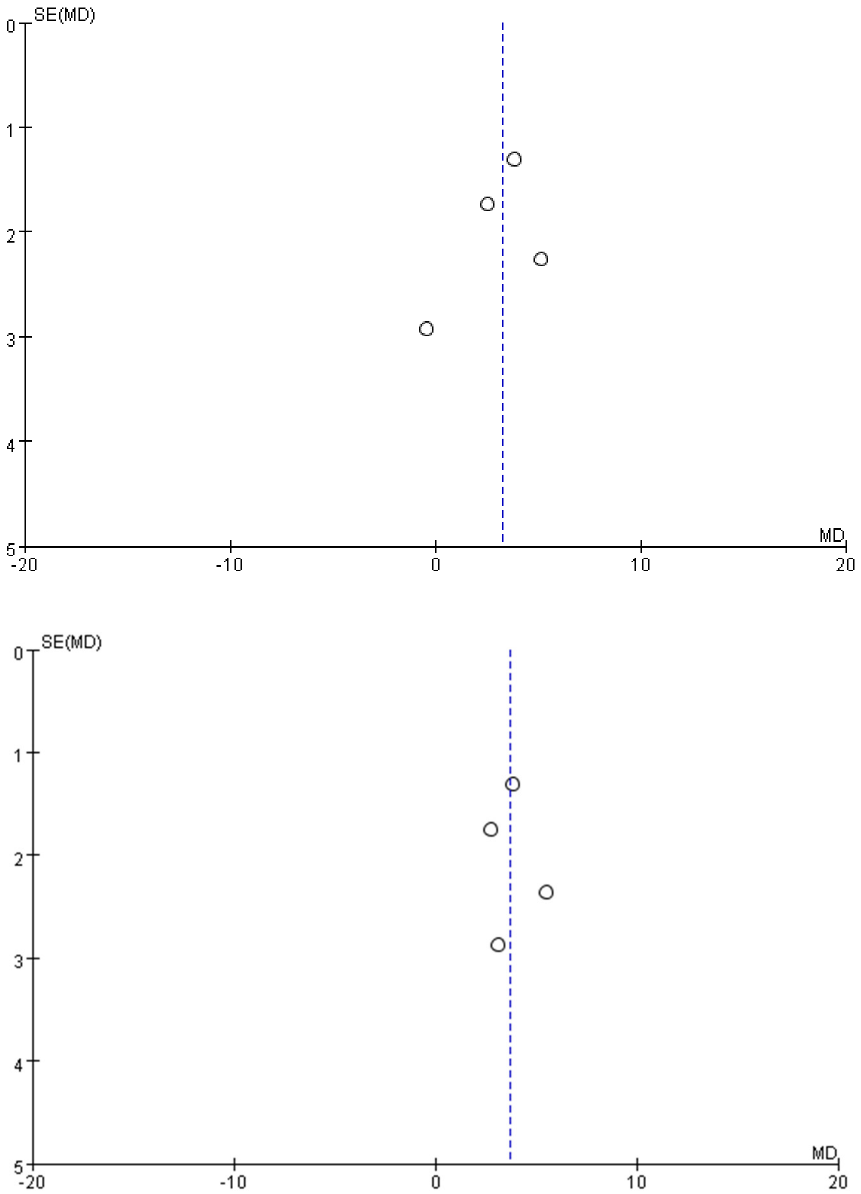

Figure S2. Funnel plot of studies comparing (a) immediate and (b) long-term FMA-distal UE between the soft robotic gloves and conventional rehabilitatio groups.

Supplementary Material S6 - Forest plots of secondary outcomes

Study or Subgroup	Experimental Mean	Experimental SD	Experimental Total	Control Mean	Control SD	Control Total	Weight	Mean Difference IV, Random, 95% CI
Kang 2020	25.92	26.95	12	26.91	27.97	11	12.7%	-0.99 [-23.48, 21.50]
Park 2021	39.91	29.55	22	33.04	27.06	22	22.1%	6.87 [-9.87, 23.61]
Shin 2016	43.1	29	24	26.4	27.3	22	23.3%	16.70 [0.43, 32.97]
Shin 2022	33.1	21.12	20	13.88	14.62	16	41.9%	19.22 [7.52, 30.92]
**Total (95% CI)**			**78**			**71**	**100.0%**	**13.34 [5.16, 21.53]**

Heterogeneity: Tau² = 5.92; Chi² = 3.26, df = 3 (P = 0.35); I² = 8%
Test for overall effect: Z = 3.20 (P = 0.001)

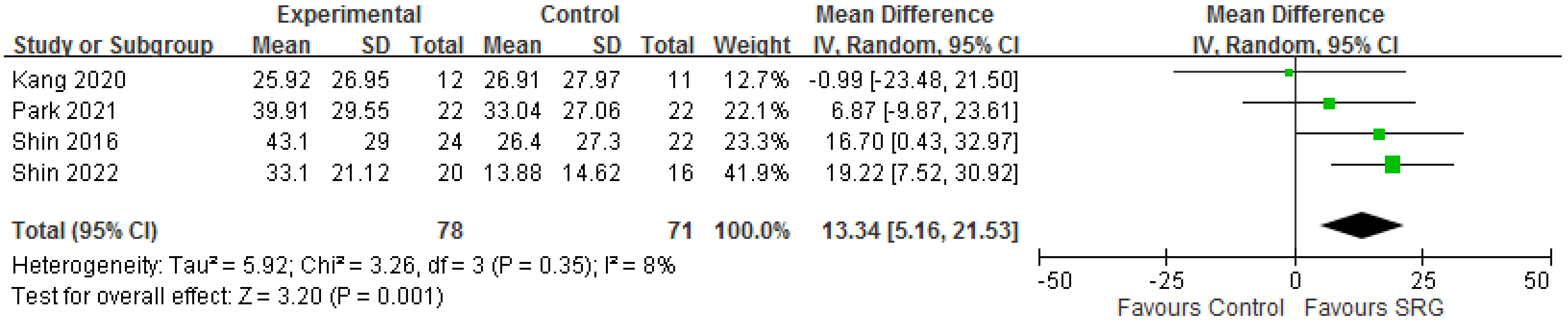

Study or Subgroup	Experimental Mean	Experimental SD	Experimental Total	Control Mean	Control SD	Control Total	Weight	Mean Difference IV, Random, 95% CI
Kang 2020	40.08	30.02	12	29.64	33.1	11	13.3%	10.44 [-15.47, 36.35]
Shin 2016	43.7	29.9	24	26.6	27.7	22	32.2%	17.10 [0.45, 33.75]
Shin 2022	40.9	23.52	20	18	15.41	16	54.6%	22.90 [10.12, 35.68]
**Total (95% CI)**			**56**			**49**	**100.0%**	**19.38 [9.94, 28.82]**

Heterogeneity: Tau² = 0.00; Chi² = 0.82, df = 2 (P = 0.66); I² = 0%
Test for overall effect: Z = 4.02 (P < 0.0001)

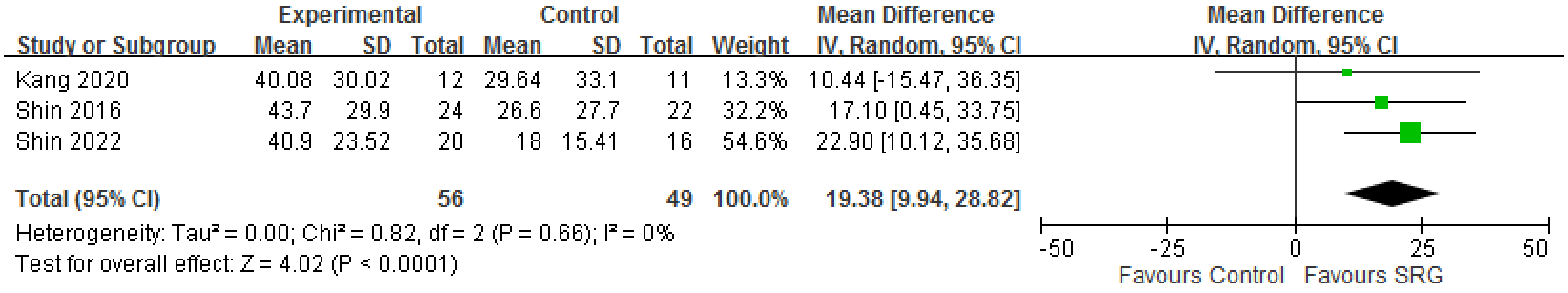

Figure S3. Mean difference (95% CI) of the (**a**) immediate and (**b**) long-term effect of soft robotic gloves on Jebsen–Taylor hand function test compared with conventional rehabilitation.

Study or Subgroup	Experimental Mean	Experimental SD	Experimental Total	Control Mean	Control SD	Control Total	Weight	Mean Difference IV, Random, 95% CI
Kang 2020	19.75	13.62	12	23.8	25.35	11	30.9%	-4.05 [-20.90, 12.80]
Park 2021	31.5	18.46	22	24.88	26.39	22	48.4%	6.62 [-6.84, 20.08]
Vanoglio 2017	19	20.635	14	13.426	32.099	13	20.8%	5.57 [-14.95, 26.10]
**Total (95% CI)**			**48**			**46**	**100.0%**	**3.11 [-6.25, 12.47]**

Heterogeneity: Tau² = 0.00; Chi² = 1.01, df = 2 (P = 0.60); I² = 0%
Test for overall effect: Z = 0.65 (P = 0.51)

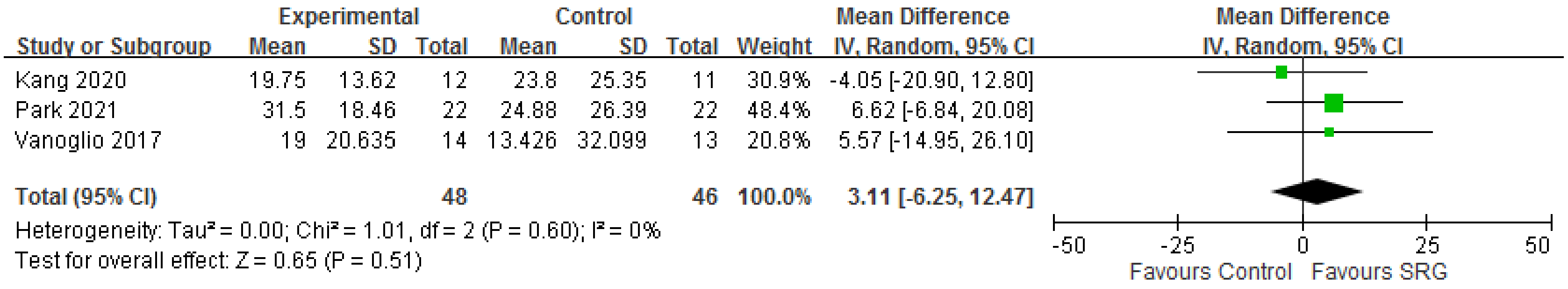

Figure S4. Mean difference (95% CI) of the effect of soft robotic gloves on grip strength compared with conventional rehabilitation.

Study or Subgroup	Experimental Mean	Experimental SD	Experimental Total	Control Mean	Control SD	Control Total	Weight	Mean Difference IV, Random, 95% CI
Carmeli 2011	32	11.6	16	31.4	16.1	15	69.6%	0.60 [-9.33, 10.53]
Kang 2020	19.08	17.14	12	22.91	19.43	11	30.4%	-3.83 [-18.86, 11.20]
**Total (95% CI)**			**28**			**26**	**100.0%**	**-0.75 [-9.03, 7.54]**

Heterogeneity: Tau² = 0.00; Chi² = 0.23, df = 1 (P = 0.63); I² = 0%
Test for overall effect: Z = 0.18 (P = 0.86)

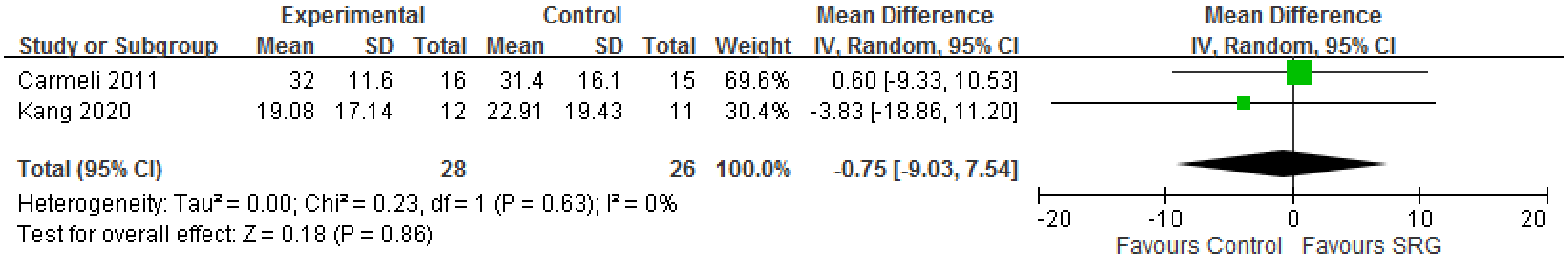

Figure S5. Mean difference (95% CI) of the effect of soft robotic gloves on box and blocks test score compared with conventional rehabilitation.

Supplementary Material S7 - Forest plots of FMA-UE baseline

Study or Subgroup	Experimental Mean	Experimental SD	Experimental Total	Control Mean	Control SD	Control Total	Weight	Mean Difference IV, Random, 95% CI
Carmeli 2011	46.8	13.1	16	49.3	9.4	15	28.0%	−2.50 [−10.49, 5.49]
Kang 2020	33.83	13.99	12	35.55	15.06	11	12.6%	−1.72 [−13.63, 10.19]
Shin 2022	41.3	8.9	20	37.13	12.84	16	32.6%	4.17 [−3.23, 11.57]
Wang 2023	9	12	23	14	16	23	26.8%	−5.00 [−13.17, 3.17]
**Total (95% CI)**			**71**			**65**	**100.0%**	**−0.89 [−5.12, 3.33]**

Heterogeneity: $Tau^2 = 0.00$; $Chi^2 = 2.94$, df = 3 (P = 0.40); $I^2 = 0\%$
Test for overall effect: Z = 0.41 (P = 0.68)

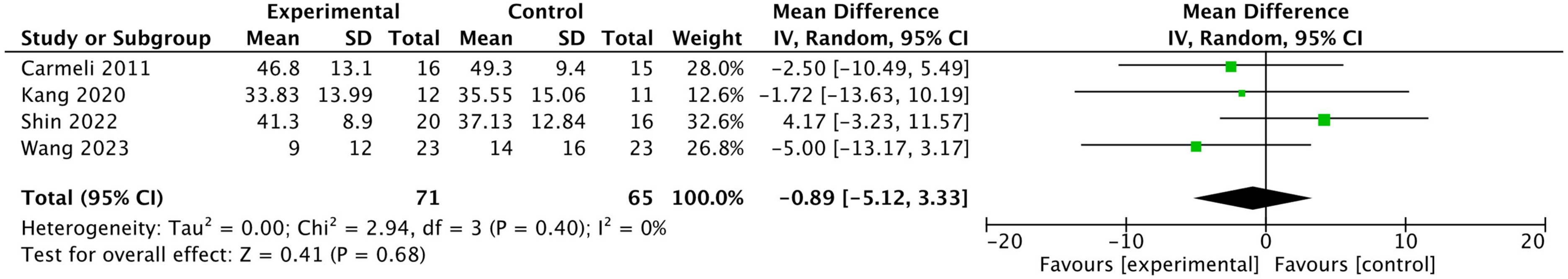

Figure S6. Mean difference (95% CI) of the baseline on FMA-UE of subacute stroke patients between groups.

Study or Subgroup	Experimental Mean	Experimental SD	Experimental Total	Control Mean	Control SD	Control Total	Weight	Mean Difference IV, Random, 95% CI
Guo 2022 a	14.9	5.23	10	14.4	8.69	10	36.2%	0.50 [−5.79, 6.79]
Guo 2022 b	14.9	7.98	10	14.4	8.69	10	26.7%	0.50 [−6.81, 7.81]
Shin 2016	53.4	8.7	24	48.2	12.3	22	37.1%	5.20 [−1.01, 11.41]
**Total (95% CI)**			**44**			**42**	**100.0%**	**2.24 [−1.54, 6.02]**

Heterogeneity: $Tau^2 = 0.00$; $Chi^2 = 1.39$, df = 2 (P = 0.50); $I^2 = 0\%$
Test for overall effect: Z = 1.16 (P = 0.24)

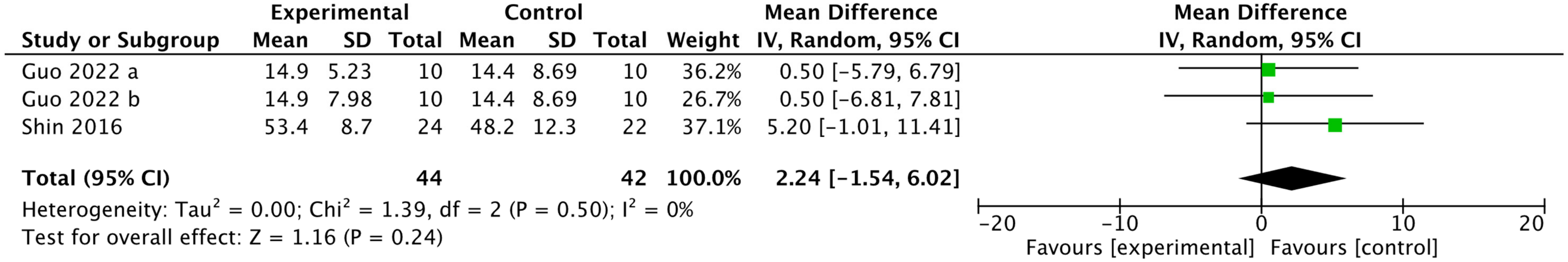

Figure S7. Mean difference (95% CI) of the baseline on FMA-UE of chronic stroke patients between groups.

		Subacute phase	Chronic phase
Mean of experimental group	Number of patients	71	44
	Baseline	30.814	35.9
	Post-intervention	41.135	43.277
Mean of the increase amount		10.321	7.377
Mean of control group	Number of patients	65	42
	Baseline	31.487	32.105
	Post-intervention	36.14	33.219
Mean of the increase amount		4.653	1.114

Figure S8. Mean of the increase amount on FMA-UE of stroke patients in subacute phase and chronic phase
